# Restoring Ravaged Heart: Molecular Mechanisms and Clinical Application of miRNA in Heart Regeneration

**DOI:** 10.3389/fcvm.2022.835138

**Published:** 2022-02-10

**Authors:** Vandit Shah, Jigna Shah

**Affiliations:** Institute of Pharmacy, Nirma University, Ahmedabad, India

**Keywords:** miRNA, heart regeneration, cardiac development, cardiomyocyte, cardiovascular diseases

## Abstract

Human heart development is a complex and tightly regulated process, conserving proliferation, and multipotency of embryonic cardiovascular progenitors. At terminal stage, progenitor cell type gets suppressed for terminal differentiation and maturation. In the human heart, most cardiomyocytes are terminally differentiated and so have limited proliferation capacity. MicroRNAs (miRNAs) are non-coding single-stranded RNA that regulate gene expression and mRNA silencing at the post-transcriptional level. These miRNAs play a crucial role in numerous biological events, including cardiac development, and cardiomyocyte proliferation. Several cardiac cells specific miRNAs have been discovered. Inhibition or overexpression of these miRNAs could induce cardiac regeneration, cardiac stem cell proliferation and cardiomyocyte proliferation. Clinical application of miRNAs extends to heart failure, wherein the cell cycle arrest of terminally differentiated cardiac cells inhibits the heart regeneration. The regenerative capacity of the myocardium can be enhanced by cardiomyocyte specific miRNAs controlling the cell cycle. In this review, we focus on cardiac-specific miRNAs involved in cardiac regeneration and cardiomyocyte proliferation, and their potential as a new clinical therapy for heart regeneration.

## Introduction

An uninterrupted supply of oxygen and nutrients by the heart is vital for the function of every cell, tissue, and organ in our body. Therefore, acquired and congenital heart diseases are a leading cause of death in developed and developing countries (1). Irreversible damage to cardiomyocytes is caused by myocardial infarction, ischemic heart disease, hypertension, and genetic mutations. Thereby, deteriorating heart functioning to heart attack and eventually death (2). Unlike, neonatal mammalian heart, adult mammalian heart regeneration is inadequate and forms scar tissue instead. Scar formation hinders the cardiac blood supply, worsening cardiac damage to heart failure (3). Current therapies and medical devices focus on symptomatic relief, however, undermines the replacement of lost cardiac muscle. Stem cell-based therapies targeted toward cardiac repair are being tested for more than a decade, showing insignificant improvement in cardiac function (4–8). Additionally, the molecular basis of stem cells based cardiac function improvement is not evident. Also, the retention of stem cells at the injured heart tissue is questionable. Thereby, suggesting the unmet clinical application of therapeutic strategies to replace cardiomyocytes and ameliorate cardiac regeneration.

Prenatal mammals possess a unique potential to regenerate their heart post-injury. But this regeneration capacity remains for a very short period following birth (9). In neonatal mammals, transitory cellular and molecular response initiated from pre-existing cardiomyocytes drive the myocardial regeneration (10, 11). Various signaling pathways like thyroid hormone signaling (12), Hedgehog (13), Notch (14), ErbB2/4 (15, 16), and Hippo/YAP signaling pathways (17, 18) are being studied as a potential for adult heart regeneration. Similarly, non-muscle cells reprogramming to cardiac muscle cell fate in the injured heart to potentially integrate and repopulate it is being investigated as an alternative approach (19–22).

**Figure 1:**
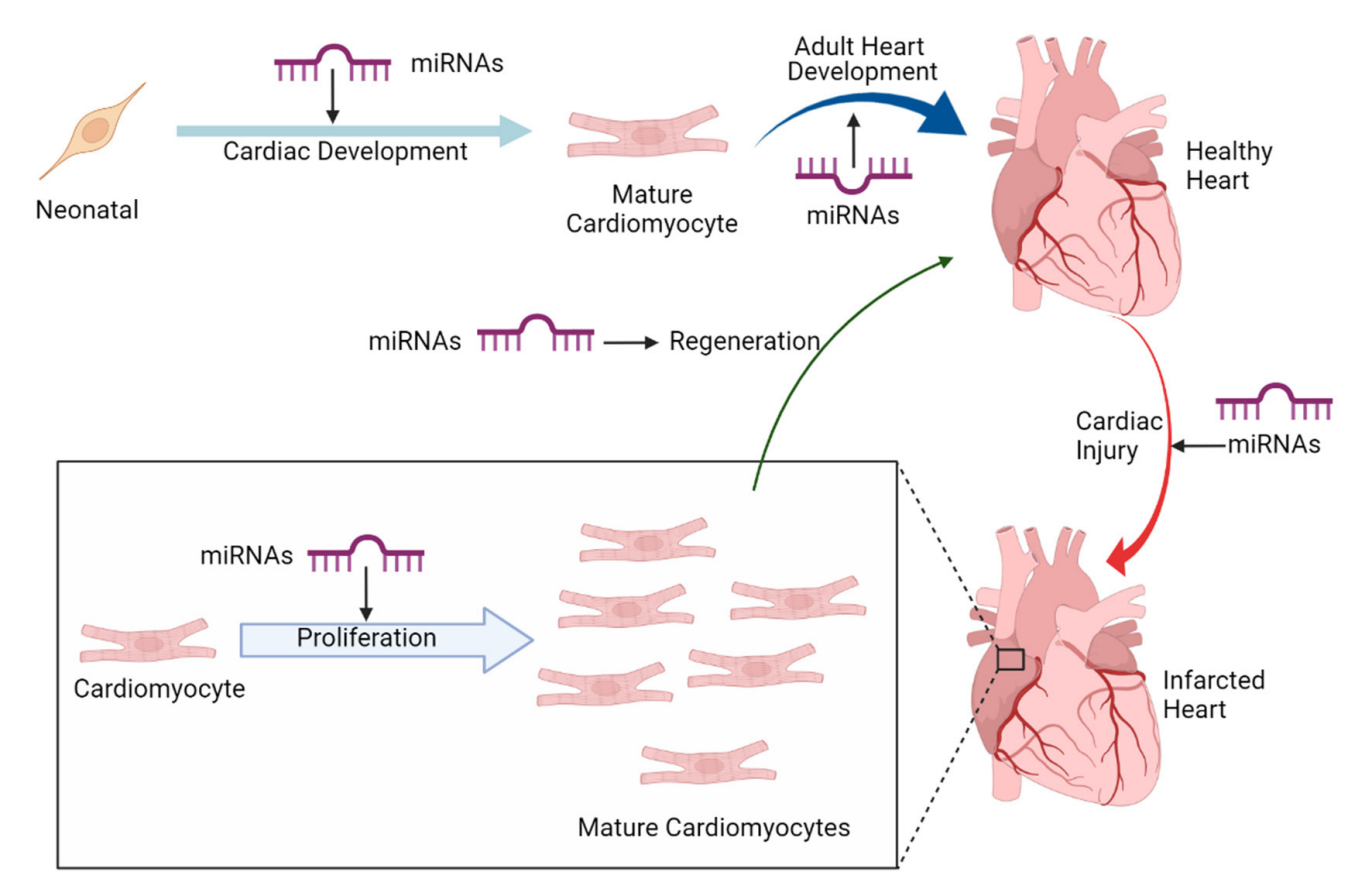
miRNAs in cardiac development and regeneration.

Recently, miRNAs (small non-coding RNAs, that regulate target gene expression post-translationally) have emerged as a potential therapeutic candidate for cardiomyocyte proliferation and heart regeneration (23). Several miRNAs have been shown to regulate and maintain cardiac cell fate specification of cardiac progenitor cells (24). Thus, studies pertaining to understand miRNA transcriptome and regulatory pathways in cardiac progenitors, regulated by miRNAs are gaining momentum. Furthermore, research in regenerative therapeutics of adult heart to develop embryonic cardiovascular progenitor miRNAs and adult cardiac-specific miRNAs based miRNA therapy is being tested. Figure 1 implicates on the role of miRNAs in cardiac development and regeneration. This review focuses on molecular regulatory mechanisms governed by miRNAs that induce cardiac regeneration and how these mechanisms can be targeted to potentially achieve adult mammalian heart regeneration.

## miRNA in Development

miRNA mechanism of action and biogenesis has been reviewed extensively, as the field has evolved over the decade (25). Briefly, miRNA controls post-translational gene expression and has been shown to play a pivotal role in embryonic development and pluripotency maintenance (26). For instance, in mice the heterozygous mutant of Oct4 showed reduced protein expression, suggesting a decrease in the stem cell pool in the embryo. Furthermore, embryonic lethality was observed in mice with homozygous Dicer 1 knockout. Thereafter, several miRNAs involved in pluripotency maintenance of the embryonic stem cells were discovered such as the miR- 290 family (miR-295, miR-294, miR-291-3p) and miR-34a (27–29). Also, miR-372 and miR-302 showed increased efficiency in pluripotent stem cell reprogramming (30, 31). Early embryogenesis entails the differentiation of different cell types from single totipotent cells, through morphogen gradients in order to transmit positional information within the embryo (32). miRNAs also play an important role in lineage differentiation and stem cell migration into different tissues and organs. miR-430 monitors the nodal morphogen gradient by acting on both the agonist and antagonist of nodal, thereby avoiding patterning mistakes in the embryo (33). miR-430 also enables degeneration of active morphogen mRNAs that are not vital such as *sdf1a* transcript is degenerated in all cells, except the cells actively transcribing new *sdf1a* (34, 35). Conversely, several tissue-specific miRNAs have been shown to promote trans-differentiation of the cells (36, 37). Furthermore, feedback and feed-forward loops between transcriptional factors and miRNAs, reinforce or counterbalance cellular decisions toward differentiation and controls cell fate (38). These findings have established the miRNAs as a promising candidate for the development of regenerative therapies.

## miRNA in Mammalian Cardiogenesis

**Figure 2:**
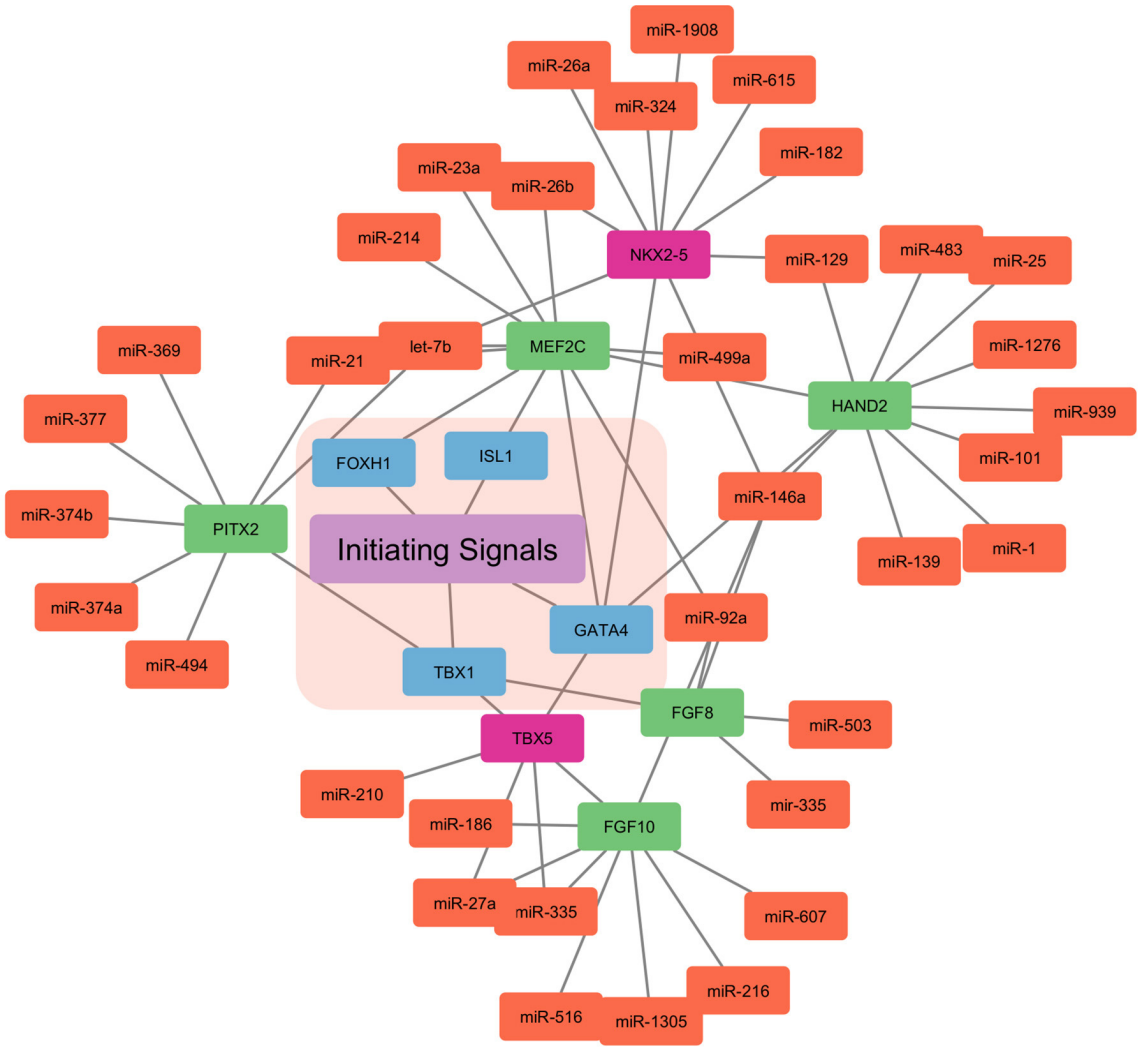
miRNA based regulation of cardiogenesis. The initiating signals, i.e., extrinsic and intrinsic signals in the primary and secondary heart field regulate the cardiogenesis. The nodes in blue represent the central transcription factor orchestrating the developmental signals. Nodes in red represent the transcription factor only expressed in the primary heart field. Whereas, nodes in green represent the transcription factor only expressed in the secondary heart field. Nodes in light coral color represent the miRNAs involved in the control of the overall cardiogenesis.

Myocardial cells (Fibroblasts and Cardiomyocytes) derived from mesoderm at gastrulation contribute toward the formation of the heart (39, 40). Early cardiac progenitors derived from the anterior mesoderm forms the primary heart field, also called the cardiac crescent. On the other hand, pharyngeal mesoderm contributes toward the development of secondary heart field between and anterior to cardiac crescent. In mice by the embryonic day 8, linear heart tube is formed by the cells migrated from the cardiac crescent in order to provide a scaffold for heart development. Furthermore, cells migrated from the secondary heart field contribute toward the development of venous and arterial poles. This leads to a beating heart tube, composed of cardiomyocytes and underlying endothelial cells. By embryonic day 8.5 primitive atria and ventricles are formed by uneven remodeling and growth of linear heart tube. Heart maturation leads to a separation between atria and ventricles by embryonic day 15. Also, positioning the outflow tract and inflow tract on the anterior pole of the heart. The right and left atria are formed by both primary and secondary heart fields. Left ventricles are formed from the primary heart field, while the right ventricle is formed by the secondary heart field (41–45). Mammalian heart development is well-orchestrated crosstalk between primary and secondary heart field, along with extrinsic and intrinsic signals. This cardiogenesis is tightly regulated by the network of signaling pathways and transcriptional regulation, which is in turn highly regulated by the miRNAs (41, 46) (Figure 2).

WNT signaling pathway from neural tube and notochord suppress differentiation into cardiomyocytes, whereas Bone morphogenetic protein (BMP) derived from endoderm enhances the cardiomyocytes specification (47). Furthermore, Pitx2 transcription factor modulates wnt signaling and miRNAs (miR-1, miR-29, and miR-200) which, in turn leads to Ca^+2^ remodeling (48). Other signals such as Notch, sonic hedgehog (SHH), Hand1/2, and FGF are involved in the downstream activation of genes encoding transcriptional activators (45, 49, 50). Thereby, inducing cardiac progenitor migration and setting up of primary and secondary heart fields. Transcription factor T-box 1(TBX_1_), centrally regulates the secondary heart field, by controlling the cardiac outflow tract development. NKX_2−5_ (NK_2_ homeobox 5) and GATA_4_ are central transcription factors in the primary and secondary heart fields. *NKX*_2−5_ and *Isl1* contribute to heart tubes, chambers, and nodes formation (51–54). These transcription factors are also cell type-specific, such as transcription factor T-box 5 (TBX_5_) is solely expressed in the primary heart field. Similarly, *Isl1* is only expressed in the secondary heart field.

miRNA machinery is co-opted to repress inhibitors of cardiac differentiation, thus increasing the expression of cardiac specific-genes during cardiogenesis. Cardiac tissue-specific *Dicer* knockout ceased the cardiac development. Also, acquired pathological abnormalities like fibrosis, biventricular enlargement, hypertrophy, and heart failure (55). Several cardiac-specific miRNAs like miR-133 and miR-1, suppress ectoderm and endoderm lineage expression and mesoderm formation (56, 57). Furthermore, miR-133 acts on cyclin D2 and serum response factors to aid cardiomyocytes proliferation (58, 59). miR-1 inhibits Delta-1 from embryonic stem cells and promotes differentiation into cardiac lineage cells (59). miR-1 targets the miRNA response element (MRE) in the 3′ UTR region of the Hand2 and Irx5, which has been shown to have a key effect on cardiac development (60). Cardiac progenitors in the second heart field express the BMP signals that co-ordinate with miR-17−92 and repress the progenitor gene expression of Isl1 and Tbx1. Thereby, promoting myocardial lineage differentiation toward the cardiac outflow tract (61). Furthermore, miR-130a targets Friend-of-GATA 2 (FOG2) and regulates cardiac development in mice (62). Other miRNAs having a vital role in cardiac development are enlisted in Table 1. Currently, there is dearth of knowledge about the miRNA involved in human cardiac development. Although studies have shown the involvement of miRNAs in heart chamber, outflow tract, and septum development (72). Our current understanding is particularly reliant on *in vitro* human pluripotent stem cell differentiation into cardiac lineage cells like cardiomyocytes, cardiac progenitors, smooth muscle cells, and endothelial cells (73). miR-1 promotes differentiation to cardiomyocytes and repress endothelial cell fate by inhibiting WNT and FGF signaling pathways via. suppressing the expression of FZD7 and FRS2, respectively (74). miR-1 also represses gene expression of Hand2, KCNJ, HDAC4, GJA1 that are crucial for cardiac function and development (75). miR-499 through SOX6 inhibition governs the differentiation of cardiac progenitor cells to cardiomyocytes. Other cardiac-specific miRNAs like miR-669, miR-204, and miR-23 are shown to enhance cardiac progenitor differentiation, however, their mechanism needs to be studied (76, 77). Therefore, precise and orchestrated expression of miRNA is viral for proper morphological and electrophysiological development of the heart.

**Table 1:** miRNAs involved in cardiac development.

**miRNA**	**Target**	**Functions**	**References**
miR-1	Hand2, Delta, Delta-1	Promotes cardiomyocytes differentiation, enhances muscle and cardiac progenitor cells expansion, cardiac lineage determination and mesoderm formation.	(59, 60, 63)
miR-15	Chk1	Cardiac cell proliferation	(64)
miR-17-92	Isl1, Tbx1, PTEN	Governs cardiac progenitor cells proliferation.	(61, 65)
miR-126	VEGF pathway, SPRED1 and PIK3R2/p85-β	Cardiac Vascularization	(66, 67)
miR-130a	Fog-2	Organizes myocardium growth.	(62)
miR-133a	Cyclin D2, Serum response factor	Regulates cardiomyocytes proliferation through negative feedback.	(58)
miR-138	Cspg2	Governs maturation of ventricular cardiomyocytes.	(68)
miR-208	Mby6, Mby7, Thrap1	Myosin expression and switching during fetal and adult stage.	(69)
miR-218	Slit2, Robo1	Cardiac Morphogenesis	(70)
miR-222	P27, HMBOX1, HIPK1	Regulates proliferation and differentiation of cardiomyocytes.	(71)

## miRNA and Cardiac Proliferation

Cardiomyocytes are the most abundant cell mass in the heart but are outnumbered by the total of endothelial cells, fibroblast, smooth muscle cells, and inflammatory cells (78). This group of cells forms a microenvironment that affects cardiac cells proliferation by paracrine signaling of the secreted molecules like growth factors, cyclins, cyclin-dependent kinase (CDKs), and cytokines and thus, are vital for cardiac repair and cell cycle reentry (79–83). Overexpression of cell cycle activators like SV40 T antigen, cyclinD, cyclinA2 promotes proliferation and dedifferentiation of post-mitotic cardiomyocytes (84–87). miR-195 has been shown to be overexpressed in cardiomyocytes, leading to cell cycle arrest at the G2 phase. miR-195 targets checkpoint kinase 1 (*Chek1*), which encodes protein kinase promoting mitotic progression and G2-M transition (64). In mice, overexpression has also led to ventricular septal defects and hypoplasia like defects, and post-myocardial infarction lead heart regeneration (64). On the other hand, anti-miRNAs having a similar seed sequence as miR-195 showed increased cardiomyocytes in the mitotic phase. The mice treated with anti-miR-195 showed improved contractile function after ischemic reperfusion injury (88). miR-199a-3p and miR-590-3p target mRNA of HOP homeobox (HOPX), its protein product suppresses cardiomyocytes proliferation. Both miRNAs also target mRNA HOMER1, whose protein product alters Ca^+2^ signaling by interacting with the ryanodine receptor (RyR). Furthermore, miR-590-3p targets chloride intracellular channel 5 (CLIC5), which is a cell proliferation inhibitor. In neonatal mice, miR-199a-3p and miR-590-3p expression in cardiomyocytes improved cardiac function and reduced fibrotic scar formation after cardiomyocytes loss following injury. miR-302-367 cluster promotes cardiomyocytes mitosis by either targeting the Hippo pathway or by upregulating the expression of homeodomain transcription factor Pitx2. Pitx2 in turn activates gene expression of ROS scavengers, Nrf2, and electron transport chain to promote cardiomyocytes mitosis (89). Mechanistically miR-302-367 targets 3′ untranslated region (3′ UTR) of LATS2 and Mst1, which are key components of Hippo signaling and regulators of cardiomyocytes proliferation (90, 91). However, the prolonged expression of miR-302-367 leads to a decrease in cardiac function and cardiomyocytes dedifferentiation (90). miR-128 deletion induce cell cycle re-entry by upregulating chromatin regulator SUZ12 (Polycomb protein SUZ12), which in turn activates CDK2 and cyclin E and repress p27 (92).

miR-214, miR-17-92 cluster, miR-222 cluster have also been shown to enhance *in vivo* cardiac repair (65, 71, 93, 94). Furthermore, cardiac-specific overexpression of miR-17-92 cluster leads to increased cardiomyocytes proliferation, heart weight, and hyperplasia without cardiac hypertrophy (65). *In vitro* studies showed miR-17-92 cluster induced downregulation of PTEN expression leads to cardiomyocytes proliferation. Anti-miR-34a in adult mice has been shown to reduce fibrosis and necrosis post-myocardial infarction additionally boosts cardiac regeneration and myocardial function (95, 96). miR-34a acts on cyclin D1, Bcl2, Sirt1, and PPP1R10 that by cardiomyocytes cell cycle arrest, apoptosis, DNA damage response, and telomerase shortening to improve cardiac function. Other several miRNAs that promote/repress cardiomyocytes proliferation and heart regeneration are listed in Table 2. These findings demonstrate the therapeutic potential of miRNAs in increasing cardiomyocytes proliferation, improving cardiac function, cell-cycle reentry, and decreasing scar formation in injured heart tissue.

**Table 2:** miRNAs that controls cardiomyocytes proliferation and heart regeneration.

**miRNAs**	**Positive control/negative control**	**Target**	**Function**	**References**
Let-7i	Negative	Ccnd2, e2f2	Overexpression leads to inhibition of cardiomyocytes differentiation and proliferation.	(97)
miR-1	Negative	Hand2, ccnd1	Overexpression suppress G1/S phase transition of cardiomyocytes.	(98, 99)
miR-19a/19b	Positive	PTEN	Overexpression or miR-mimics leads to enhanced cardiac regeneration post-myocardial infarction. Also stimulates cardiomyocytes proliferation.	(65)
miR-25	Positive	Bim, FBXW7	miR-mimics leads to cardiomyocytes proliferation in both neonatal and adults.	(100, 101)
miR-34a	Negative	Bcl2, ccnd1 and sirt1	Inhibition leads to increase in cardiomyocytes proliferation and improves recovery post-myocardial infarction.	(96)
miR-133a	Negative	Srf and ccnd2	Loss of expression leads to aberrant cardiomyocytes proliferation and ectopic expression of smooth muscle genes in heart.	(58)
miR-195	Negative	Chek1	Overexpression leads to ventricular septal defects and ventricular hypoplasia.	(64)
miR-204	Positive	Jarid2	Enhance cardiomyocytes proliferation throughout embryonic and adult stage.	(102)
miR-216a	Negative	Jak2	Overexpression inhibits cardiomyocytes proliferation, whereas inhibition stimulates cardiomyocytes proliferation.	(103)
miR-294	Positive	Wee1	Overexpression leads to cell cycle re-entry and cell cycle activity. Thereby, enhancing heart function.	(104)
miR-302-367 cluster	Positive	Hippo signaling pathway	miR-mimics promotes cardiac regeneration post-myocardial infarction. Also improves cardiac function.	(90)
miR-590-3p	Positive	Homer1, Hippo signaling pathway.	Overexpression leads to cardiomyocytes proliferation and reduces scar area.	(105, 106)
miR-708	Positive	Mapk14	Positively regulates cardiac function and regeneration	(107)
miR-1825	Positive	Hippo signaling pathway	Overexpression leads to improved heart function and robust cardiomyocytes proliferation.	(108)

## miRNA in Cardiovascular Disorders

miRNAs disturbance associated with cardiac development and progenitors leads to structural abnormalities of the heart. Several miRNAs associated with cardiogenesis and cardiac progenitors showed dysregulation in congenital heart defects. Whereas, adult heart-specific miRNAs are co-related with cardiovascular pathologies like myocardial infarction, ventricular hypertrophy, and arrhythmias (109–111). miRNAs are also being investigated as a diagnostic biomarker to detect cardiac diseases and understand disease pathology (112). Defects in cardiomyocytes proliferation and migration pathways are associated with congenital heart disease. miRNA transcriptome shows distinct expression of miRNAs in different pathological conditions, suggesting dysregulation of the pathways. Several studies depicts disease specific distinct miRNAs expression. For instance, low expression of miR-30b, miR-103, miR-142-3p, miR-342-3p is observed in heart failure patients as compared to chronic pulmonary obstructive disease (113). Further several studies stated distinguishing cardiac pathologies like dilated cardiomyopathies, ischemic cardiomyopathy, and aortic stenosis based on miRNAs levels. Ikeda et al. showed 43 differentially expressed miRNAs, of which at least one miRNA was distinct to a specific disease group (114). mRNA expression profile when compared with miRNA expression could provide a promising diagnostic tool to differentiate diverse cardiac pathologies. Cardiac disease-specific miRNAs identification also provides a platform for a new drug target which, can be harnessed to treat currently incurable heart disease. A list of miRNAs, their expression profile in cardiomyocytes, along with their targets, and associated cardiovascular diseases are listed in Table 3.

**Table 3:** Cardiovascular disease associated with miRNAs.

**miRNAs**	**Expression profile**	**Target**	**Disease condition**	**References**
miR-1	Underexpressed	Mef2a, Hand2, SOX9	Cardiac hypertrophy, Ventricular septal defect.	(60, 74, 115)
miR-21	Overexpressed	unknown	Myocardial Infarction	(111)
miR-23a	Overexpressed	Foxo3a	Cardiac hypertrophy	(110)
miR-26b	Overexpressed	GATA4 Cox5a	Heart failure Myocardial infarction	(116, 117)
miR-29b	Overexpressed	Unknown	Myocardial Infarction	(111)
miR-30c	Underexpressed	ATG5	Heart failure with preserved ejection fraction.	(118)
miR-126	Underexpressed	PI3KR2a	Faulty angiogenesis	(119)
miR-133	Overexpressed	Bmf, Bim	Ventricular tachycardia	(120)
miR-146a	Underexpressed	TRAF6	Heart failure with preserved ejection fraction.	(118)
miR-150	Underexpressed	EGR1	Atrial fibrillation Myocardial fibrosis	(121) (122)
miR-181	Overexpressed	BMPR2	Ventricular septal defects	(74)
miR-195	Overexpressed	CHECK1	Ventricular septal defects	(64)
miR-328	Overexpressed	CACNA1c, CACNb2	Atrial Fibrillation	(123)
miR-421	Overexpressed	SOX2	Tetralogy of fallot	(124)
miR-483	Overexpressed	Igf1	Atrial fibrillation	(125)
miR-499	Overexpressed	Mef2	Myocardial infarction	(126)
miR-1254	Overexpressed	Smurf1	Chronic heart failure	(127)

In mice, overexpression of miR-21 has shown to reduce apoptosis and myocardial infarction by 36.9%. miR-21 also enhances cardiomyocytes proliferation and viability post-myocardial infarction by targeting PTEN and the Akt pathway (128). Circulating miRNAs are widely studied to harness the potential of biomarkers as a risk assessment tool for heart failure. About 30 differentially expressed miRNA has been identified for heart failure, miR-199a, miR-27a, miR-26b, miR-18a, miR-652, miR-30, and miR-106a are significantly less expressed in patients with heart failure. Also, miR-1 is downregulated and miR-21 is upregulated in patients with symptomatic HF (129). miR-210 levels are directly correlated with the severity of heart disease. Inversely, patients recovering from heart failure show a decrease in miR-210 levels (130). Furthermore, miR-423 negatively correlates while, miR-208b, miR-499, miR-1306, and miR-1254 positively correlate with the mortality of heart failure patients (127, 131–133).

Plasma levels of miR-21 are significantly higher in patients with acute myocardial infarction (AMI). Additionally, miR-21 levels are correlated with creatine kinase and cardiac troponin I (cTnI) that are traditional biomarkers for AMI (111, 134). Similarly, miR-208 upregulation was observed in patients with AMI and had a high mortality rate within 6 months (135, 136). Post-cardiomyocyte injury, the high levels of miR-499 are highly associated with cTnI in patients with AMI (137). The combination of miR-1, miR-208, and miR-499 has significantly higher predictive value for AMI patients as compared to conventional biomarkers (138).

miR-150 levels were observed to be reduced by 3.2 folds in atrial fibrillation patients, affecting pathways associated with fibrosis, platelet function, and inflammation (121, 139). Dysregulation of miR-29 and miR-208b is observed in patients with atrial fibrillation (AF). Their downstream targets involve genes implicated in apoptosis and cardiac fibrosis (140–142). Also, miR-328 controls atrial remodeling by acting on Ca^+2^ channel protein expression, specifically L-type Ca+2 channels α1c (CACNA1C) and β1 (CACNB2) subunit (123, 143). Similarly, upregulation of miR-21 in atria affects the mitogen-activated protein kinase pathway. Thereby, governing the level of cardiac hypertrophy and interstitial fibrosis (144).

## Clinical Application Potential of miRNAs

miRNAs by the virtue of their small size, ability to control the expression of various mRNA, and relatively pleiotropic effects have emerged as a promising therapeutic candidate to treat cardiac diseases. Besides post-translational silencing by miRNAs, gene expression can be altered by other methods like epigenetic modification, inhibition or degradation of translated protein, decreasing transcription factor levels, and entire gene knockout (145–149). However, drugs inhibiting and degrading transcription factors or translated protein are small molecules, which require large-scale screening to check the efficacy (150). Additionally, certain protein targets are non-druggable, i.e., without small molecule interaction specific binding pockets (151). Targeted gene mutagenesis with CRISPR system is into clinical trials but the field is very naive and requires supportive data (152). Small molecules used for epigenetic modification of DNA are typically unspecific in their interaction and tend to cause global modification of the target cell's genomic DNA. miRNAs are versatile and provide a transient control over gene expression. Since the last decade, several cardiac specific miRNAs have been elucidated. Once miRNAs have been identified, complementary oligonucleotide sequence synthesis is trifling to develop drugs for treatment. Currently, miRNA based drug substances are based on locked nucleic acid (LNA) to develop antisense oligonucleotide for target mRNA sequence, thereby silencing target gene (153). A large number of miRNAs regulatory target predicting databases have emerged in the last decade (154, 155). Several independent algorithms like TargetScan that predict miRNA binding sites based on seed regions, that are critical for protein-coding mRNA binding and its associated biological networks are developed. Other databases like Kegg and Ingenuity pathway analysis are being used for disease state and putative biological pathways. Furthermore, *in silico* programs that estimate free energy between RNA sequences, lower free energy suggests stable and strong binding (156, 157).

miRNA based therapeutics can be beneficial by inducing differentiation, proliferation, and migration of terminally differentiated cardiomyocytes. Reversal of myocardium undergoing mitotic arrest toward proliferative progenitor cells can enable tissue repair and repopulation. MGN-1374 is an anti-miR of the miR-15 family seed region. It stimulates cardiomyocyte proliferation of post-myocardial infarction heart (158, 159). Cardiac specific miRNA like miR-208 has been shown to be critical for heart failure as it prevents myosin switching and improves cardiac function by targeting MED13 (Metabolic regulator and insulin sensor). Anti-miR-208 potential as a therapeutic option is being investigated for heart failure (159). Wills tumor gene 1 (Wt1) expression in the epicardial layer promotes proliferation, differentiation into cardiac lineage cells, and neovascularization (160–162). Post-myocardial infarction, Wt1+ epicardial cells undergoes epithelial to mesenchymal transition (EMT) to enable cardiac repair (119). Let-7 miRNA expression inhibition has been linked with increased expression of EMT-related genes in epicardial cells (163). Thus, can be a promising therapeutic candidate to stimulate cardiac regeneration by providing a proliferative niche in the infarcted heart.

Targeted delivery of miRNAs to the cardiac tissue needs to overcome certain barriers such as nuclease degradation, endothelial barrier, cell membrane transfer, and minimal toxicity. Certain delivery systems like viral lipid based delivery systems are under validation. Viral-based delivery, particularly with Adeno-Associated Virus (AAV) is studied extensively. AAV9 based delivery of hsa-miR-590 and has-miR-199 showed stable expression with increased cardiomyocytes proliferation in mice heart (106). Similarly, lipid based delivery of miR-199 was able to activate cardiomyocyte proliferation and provide robust cardiac regeneration in mice (164). Both the delivery system has some limitations that need to be addressed before their clinical application. For instance, the viral delivery system prohibits single mature miRNA specific expression and the long term persistence of viral vector in transduced cardiomyocytes leads to compounding of overall therapeutic effect. Thus, leads to several unwanted downstream consequences (23, 90). On the other hand, lipid based system particle size is comparatively larger (>1 μm) that has been linked to severe toxicity and inflammation in animal studies (165). Exosomes and biocompatible injectable hydrogels-based delivery of miRNAs *in vivo* have shown efficient transduction in cardiomyocytes (108, 166, 167). Taking into account that most of our current understanding is based on small animals in which miRNA delivery is quite easy as well as their cardiomyocyte biology and cardiac physiology are markedly distinct from humans. Additional preclinical studies elucidating the appropriate treatment dosage, location, and duration in models, which represent human physiology are warranted to enable the clinical application. Although no miRNA based drug candidate has entered clinical trial phase 3 in clinicaltrials.gov database, there are several clinical trials in early phase trials. Clinical trial for LNA-modified antisense oligonucleotide (MRG 110) that antagonize miR-92 is under study for heart failure (Clinicaltrials.gov). Overall, these studies and reports indicate a promising future of miRNA-based therapeutics for cardiac diseases.

## Conclusion and Future Perspective

The end goal of restoring ravaged heart is complete heart regeneration through cardiomyocytes renewal, scar reduction, and neovascularization. Cardiac regeneration is governed by a network of complex and strictly controlled processes. Studies pertaining to the role of the regulatory network and signaling pathway critical for cardiac development have resulted in novel strategies to induce cardiac repair and regeneration. Central to this development is the miRNAs based gene regulation, which has heralded next-generation *in situ* regenerative therapies for the heart. miRNA inhibitors and mimics are easily synthesized and delivered by viral and non-viral transfection methods in small animals. Current strategies for cardiomyocytes proliferation are vastly inefficient and have been primarily tested in rodents. Therefore, preclinical trials on large animals, organoids are vital to demonstrate safety and efficacy of the therapeutic strategies. Also, with the advent of single-cell sequencing techniques, characterization of cell type specific function and expression of miRNAs will further enhance our understanding. In near future, the combination of developmental regulatory mechanism and cellular transplantation along with artificial matrices and decellularized tissue scaffolds can drive toward successful adult heart repair and regeneration.

## Author Contributions

VS conceptualized, formal analysis, writing—original draft, and writing—review and editing. JS conceptualized, supervision, and writing—review and editing. Both authors contributed to the article and approved the submitted version.

## Conflict of Interest

The authors declare that the research was conducted in the absence of any commercial or financial relationships that could be construed as a potential conflict of interest.

## Publisher's Note

All claims expressed in this article are solely those of the authors and do not necessarily represent those of their affiliated organizations, or those of the publisher, the editors and the reviewers. Any product that may be evaluated in this article, or claim that may be made by its manufacturer, is not guaranteed or endorsed by the publisher.
